# Because they’re worth it? A discussion paper on the value of 12-h shifts for hospital nursing

**DOI:** 10.1186/s12960-022-00731-2

**Published:** 2022-05-07

**Authors:** Chiara Dall’Ora, Ourega-Zoé Ejebu, Peter Griffiths

**Affiliations:** 1 Innovation Centre, NIHR ARC Wessex, Southampton Science Park, 2 Venture Road, Chilworth, Southampton, SO16 7NP UK; 2 School of Health Sciences, University of Southampton, University Road, Southampton, SO17 1BJ UK

**Keywords:** Economics, Workforce, Nursing, Productivity, Efficiency, Recruitment, Retention, Shiftwork

## Abstract

The organisation of the 24-h day for hospital nurses in two 12-h shifts has been introduced with value propositions of reduced staffing costs, better quality of care, more efficient work organisation, and increased nurse recruitment and retention. While existing reviews consider the impact of 12-h shifts on nurses’ wellbeing and performance, this discussion paper aims to specifically shed light on whether the current evidence supports the value propositions around 12-h shifts. We found little evidence of the value propositions being realised. Staffing costs are not reduced with 12-h shifts, and outcomes related to productivity and efficiency, including sickness absence and missed nursing care are negatively affected. Nurses working 12-h shifts do not perform more safely than their counterparts working shorter shifts, with evidence pointing to a likely negative effect on safe care due to increased fatigue and sleepiness. In addition, nurses working 12-h shifts may have access to fewer educational opportunities than nurses working shorter shifts. Despite some nurses preferring 12-h shifts, the literature does not indicate that this shift pattern leads to increased recruitment, with studies reporting that nurses working long shifts are more likely to express intention to leave their job. In conclusion, there is little if any support for the value propositions that were advanced when 12-h shifts were introduced. While 12-h shifts might be here to stay, it is important that the limitations, including reduced productivity and efficiency, are recognised and accepted by those in charge of implementing schedules for hospital nurses.

**Supplementary Information:**

The online version contains supplementary material available at 10.1186/s12960-022-00731-2.

## Introduction

In recent years, shifts of 12 h or longer to deliver 24-h healthcare have become increasingly common in hospitals in many countries around the world, including in Europe and the US [1]. Twelve-hour shifts arose in the 1970s and led to a change from the traditional shift system with three 8-h shifts to a shift system with two long shifts. Twelve-hour shifts were welcomed by many as an intervention that would bring value to healthcare systems, nurses and their patients [2], by reducing staffing costs; allowing a more efficient organisation of care throughout the day; increasing quality of care; improving nurse recruitment and reducing staff turnover. In this this discussion paper, after introducing the value propositions, we analyse the available evidence to support or refute these propositions.

Long shifts appeared as a solution at a moment where nursing staff was in high demand but in short supply, and maintaining safe staffing levels was challenging for healthcare systems [3]. By reducing overlaps between shifts, there were fewer nurse hours per day to be rostered. When wards operate a three 8-h shifts system, there are overlaps of between one and three hours between shifts, and three handovers across 24 h. This translates into between 26 and 30 h of staffing, and 14% of paid time covering handovers. With 12-h shifts, there are only two handovers across 24 h, usually lasting 30 min, so that staffing is needed only for 25 h. While there were no economic benefits for nurses who would still work the same weekly hours, and therefore receive the same salary, reorganising the 24-h day with two shifts would lead to financial savings for healthcare systems. Estimates from early studies indicated savings of 10 nursing hours per day in a 32-bed unit when registered nurses worked 12-h shifts compared to 8-h shifts [3].

Early reports generally concluded that the quality of care was also improved, although empirical evidence offered at the time was mostly referring to higher continuity of care due to patients seeing the same nurse throughout the day [4, 5]. Benefits were attributed to lower risk of information being lost or miscommunicated during handover, and improved staff morale. These long shift patterns rapidly became the norm in the US and were later introduced in the UK with the same assumptions: “to increase continuity [of care] and reduce costs”, as well as improving systems’ productivity [6].

While defining productivity in healthcare is challenging [7, 8], traditionally, a system’s productivity is calculated by measuring how much output is produced from the available resources (inputs) [9]. Efficiency relates to how such resources are used, with the goal of reducing waste in inputs. Similarly to productivity, efficiency in healthcare is not clearly defined [10], and productivity and efficiency are often used interchangeably [11, 12], with nursing often being viewed as a cost-centre for healthcare systems rather than generating value through the care it provides [13]. This, coupled with chronic underfunding of healthcare systems, has led many to conceptualise productivity in healthcare as doing “more with the same”, and efficiency as “the same with less” [14].

A further major challenge managers in healthcare systems are currently faced with is that of recruiting and retaining nursing staff, especially registered nurses [15–17]. When 12-h shifts were first introduced, a major supporting argument was their ability to attract and retain nursing personnel, because of the intrinsic benefits for employees. These include higher number of days off, and, as a result, better work-life balance, reduced travel and parking costs, better opportunities to spend time with family and friends, and lower childcare costs [2, 6, 18–20].

Notwithstanding the value propositions that 12-h shifts would offer, there is inconsistent and contradictory evidence on the effects of 12-h shifts on nurses’ wellbeing and job performance. Some early reports from the US indicated that nurses working long shifts experienced lower emotional exhaustion and were more satisfied with their schedule and their job in comparison to nurses working short shifts [21]. In contrast, several large observational studies point to adverse effects on both quality of care and staff outcomes [22, 23]. Such evidence leads many to question whether 12-h shifts should be adopted routinely. On the other hand, the widespread use of 12-h shifts may be a sign that these shifts are valued by healthcare managers or preferred by some nurses.

If such shifts continue to be widely used despite evidence that indicates possible harm, it is important to understand whether the perceived ‘value’ that is associated with this working pattern is realised. Therefore, in this paper, we will consider the potential value of long shifts in hospital nursing in terms of reduced staffing costs for the healthcare provider, efficient organisation of care, improved quality of care, and increased nurse recruitment and retention. Drawing from literature and search strategies from our previous reviews on shift patterns [23, 24], we expanded our search strategy for this current paper. Searches were updated in February 2022. Using Econlit, CINHAL and Medline, we combined terms pertaining to shift patterns, hospital nursing and outcomes related to value as reported in Additional file 1. While we have attempted to be explicit about our approach and have drawn on other reviews that have used more formal and comprehensive approaches to finding and selecting literature, this discussion is not based on a comprehensive review of the literature.

### Reduced staffing costs

The empirical evidence relating to 12-h shifts and staff deployment and associated costs is very limited. If, as is claimed, 12-h shifts mean that nurse-to-patient ratios can be maintained with fewer overall nursing hours per day, one would expect to see reductions in nursing hours per day associated with increased use of long shifts. Nonetheless, the direct evidence, limited to a single-site study of nurses’ rosters, found that on wards where higher proportions of 12-h shifts were deployed daily, there was no reduction in nursing hours worked per day. In addition, there was no reduction in nurse staffing costs per patient day when higher proportions of nurses worked 12-h shifts. Wards using a mixed shift system with different shift lengths had higher staffing costs, possibly because the mix of short and long shifts necessitated more, not fewer handovers [25]. While there may be little direct evidence of direct costs, other factors that may influence effective staff deployment and costs may be affected by 12-h shifts, including sickness absence. Higher sickness absence leads to increased wage bills as both the staff member and any replacement, usually more expensive workers from an external agency [26], must be paid.

When studies use objective shift and absence data linked to payroll and longitudinal methods, higher proportions of 12-h shifts are associated with higher sickness absence [21], These are studies with samples ranging between 1944 [27–29] and 38,699 nurses [29] in England, Denmark and Finland. In one study, authors found that 6 months after 12-h shifts were introduced, in a typical ward with 30 nurses working full-time, there would be approximately 12 extra hours of sickness absence per week, compared to 8 h of sickness absence per week under an 8-h shift system [28]. Nevertheless, a survey found that nurses working 12-h shifts reported missing fewer shifts due to sickness yet when cross-referencing the subjective responses to payroll data, no differences in sickness absence rates between nurses working 8- and 12-h shifts were found [21].

Nurses might experience higher sickness absence when working long shifts as the additional days off resulting from the compressed working week might not be enough to recover. Resorting to sickness absence to recover from acute fatigue would be best captured by short-term sickness absence episodes, and long shifts have been associated with both short and long-term sickness absence [27].

In summary, the value proposition that 12-h shifts lead to reduced direct staffing costs has not been supported by evidence. While the absence of evidence does not indicate that the opposite is true, the evidence that there is an association with increased sickness absence means that there is a mechanism by which staffing costs might be increased rather than decreased when long shifts are worked. More robust economic evaluations are required to shed light on the cost implications of different shift lengths.

### Efficient organisation of care

Despite the challenge of defining efficiency in healthcare, the widely researched concept of missed care in nursing provides a means of exploring the impact of shift patterns on the efficient organisation of care. Missed nursing care, also referred to as unfinished care, rationed care [30] or care left undone [31] refers to care that was deemed necessary but was not completed during a worked shift. This could include not administering medication at the right time, not monitoring patients’ vital signs, and failing to talk to patients about their care. While some level of care omission may be inevitable, if long shifts improve efficiency, the rate of missed care should be unaffected for a given staff-to-patient ratio, or reduced if total hours are the same, assuming other inputs are unchanged. Contrary to this expectation, research drawing on a survey of more than 30,000 nurses across 12 European countries found that after controlling for the patient-to-nurse ratio, higher rates of care activities left undone were reported by nurses working 12-h shifts, compared to nurses working 8-h shifts [1, 32]. When considering an objective measure of missed care—i.e. compliance with a vital signs monitoring protocol, no improvement in compliance was found for registered nurses working more 12-h shifts than their counterparts (controlling for total hours) [33].

Nonetheless, the association between long shifts and missed care is at least plausible, because nurses might need to pace themselves to maintain energy throughout the shift. Nurses may slow the level of activity down as the shift progresses, either as a direct result of fatigue or as a deliberate countermeasure to manage rising fatigue. This has been reported by nurses who, after 12-h shifts were implemented, found they were leaving some care activities incomplete because of the intensity of work over an extended period of time [34]. A further countermeasure might be increasing the proportion of break length during the shift. A pilot study of 24 nurses found that after 12-h shifts had been introduced, the proportion of break time (as opposed to total break time which is expected to increase with longer shifts) had increased compared to that during 8-h shifts [19]. Similarly, in a time-and-motion study of 10 wards, it was found that the amount of direct patient care had reduced under 12-h shifts, as demonstrated by nurses having more unofficial breaks during the shift [35]. Such time-and-motion studies would provide valuable evidence around the actual productivity of nurses under different shift systems, but there is a dearth of such studies in the current research landscape.

Recent studies have explored the effect of long shifts on what has been termed “ancillary nursing work” [36]. The term refers to activities that cannot be classified as “direct” patient care, and therefore can easily be overlooked and dismissed as ‘unproductive’, and so are often targeted for efficiency savings. There are numerous examples where a focus on proportion of direct care time as a measure of productivity reinforces this perception [37]. While emotional intellectual and organisational activities are key dimensions of nursing work, in a context of understaffing and under resourcing, nursing productivity might be misrepresented as direct patient care only [38]. This is of relevance to 12-h shifts because additional staff during the overlap between shifts is, therefore, assumed to be unproductive. While some overlap for a handover may be necessary ancillary work, any time above this is assumed to have no additional value and so there is assumed to be no detriment when one handover is eliminated. Indeed, as previously noted, eliminating a handover is potentially beneficial as each handover is an opportunity for vital information to be lost.

The available evidence suggests that overlaps between shifts may have provided useful opportunity for a range of ancillary work including staff education and continuous professional development and discussion with colleagues about patients. Such activities are potentially crucial to the delivery of safe and effective care and so the assumption that they are without value and that the time is simply unproductive is, at best, questionable [39]. Two cross-sectional surveys found that nurses working 12-h shifts reported reduced access to continuing educational opportunities and to less opportunity for discussions around patient care with colleagues compared to those working 8-h shifts. Rather than reducing information loss by eliminating a handover, nurses working long shifts were more likely to report that important patient information was being lost during handovers [39, 40]. While the ultimate value of such work for safe and effective patient care should not be assumed, nor should the opportunity for staff to discuss care and to engage in professional development be readily dismissed as unproductive.

In summary, removing the overlap and handover between shifts that results from introducing 12-h shifts does not appear to lead to a more efficient organisation of care through the day, with nurses possibly reducing their pace to save energy to manage until the end of the shift. A few studies indicate that opportunity to complete potentially valuable ancillary work is reduced under 12-h shift systems.

### Improved quality of care

The implementation of long shifts has led many to question the impact they would have on nurses’ cognitive and task performance during the shift. Besides increasing fatigue levels [41], working consecutive 12-h shifts has been associated with sleepiness and reduced sleep times [42–44], and performance and safety might be consequently affected. Just a decade after 12-h shifts had been widely adopted in the US, studies emerged to examine how the quality of care had been affected [4]. A meta-analysis of five studies found that the risk of making an error was significantly higher for nurses working 12-h shifts than for those working less than 12 h. Error was defined as self-reported error in three studies, and in two studies as nurse-reported frequency of adverse patient outcomes, including complaints from patients and family [45].

Recent studies have used more objective measures, but have generally only reported on the performance of nurses working 12-h shifts (with no comparison) [46, 47] and thus, are largely uninformative, although findings do suggest increasing errors and reduced cognitive performance over the course of consecutive long shifts [47, 48]. A single pilot study compared cognitive errors in 28 nurses working 8 or 12-h shifts finding no statistically significant difference in cognitive performance [49]. Overall, while increased performance impairments when nurses work 12-h shifts are plausible due to their impact on fatigue and sleep, the lack of objective shift and outcomes data means the current evidence is weak.

A further value proposition resulting from removing a handover during the day is higher continuity of care due to patients seeing the same nurse throughout the day. The evidence around this is, however, mixed. In some qualitative studies, nurses report higher continuity of care and better communication with patients when working long shifts [39, 50]. In contrast, some nurses report that continuity of care decreased with 12-h shifts because they are away from work for longer due to having more days off [24]. These mixed views are echoed in quantitative studies which did not find any specific shift length to enhance or decrease continuity of care [36, 51]. The methods used make it hard to distinguish the facets of continuity being reported, although it appears that perceptions may vary depending on whether continuity within a working day versus between days is the focus.

In summary, while some nurses report that long shifts facilitate continuity of care, this is not supported by observational studies of associations and no empirical data indicate that long shifts lead to better quality of care in terms of reduced errors.

### Increased recruitment and retention

Despite the frequency with which the claim of improved recruitment and retention is made, we often found chains of citation where papers cited others in support of the assertion, which in turn cited other sources with none providing substantive empirical evidence. For some examples, see [18, 40, 52, 53]. The available evidence instead focuses on perceptions of small samples of nurses already employed in a setting who pilot 12-h shifts [54]. Quantitative evidence concluded that in units with 12-h shifts there was a lower nurse vacancy rate, but a higher nurse turnover rate [21]. Such a finding would be consistent with a positive effect on recruitment, but a negative one on retention, although it would be wrong to make too strong an inference from such limited evidence, and differences in recruitment and retention might have to do more with the nature of the nursing work in different units.

A major limitation of existing research is that studies fail to report how long 12-h shifts had been introduced for prior to their evaluation, and whether the implementation had been supported or even requested by the nursing personnel. This is important because responding to staff preferences and choice when it comes to shift patterns may play an important role in recruitment and retention [55]. For instance, some discrete choice experiment studies have focused on nurses’ job preferences and revealed their preferences for flexible shift patterns, among other workplace characteristics [56–58]. Our recent literature review found that a crucial aspect in determining the success of long shifts was buy-in and support from the workforce. When these shifts were introduced as mandatory or as a blanket hospital-wide intervention, nurses were less likely to stay in their job [24]. It is also possible that positive results arise in an early honeymoon period when the staff who expressed a preference are retained, without reflecting the sustained effect or preference either in those who continue in the workforce or for new staff [59].

Although there is no direct evidence relating to staff turnover, a number of studies have investigated the impact of long shifts on nurses’ expressed intention to leave, which is a strong predictor of turnover [60, 61]. Large observational studies found that those working 12-h shifts are more (not less) likely to express an intention to leave, although the designs used cannot discount selection effects whereby those with higher turnover intention may be more likely to choose to work longer shifts [62–64].

An imbalance between organisational demand and nurse preferences when organising shift patterns may lead to unintended consequences in the longer term. The evidence on nurses’ ability to choose and negotiate their shifts is mixed. In some instances, nurses have reported having either complete or some degree of autonomy when choosing shift patterns [65]. However, in some instances, long shifts are implemented at an organisational level based on service demands rather than staff needs and preferences [28, 66, 67]. In contrast, the possibility to have choice and flexibility around shift patterns is not only valued by nurses, but also contributes to enhancing their health and wellbeing [68, 69], and possibly recruitment and retention, although the latter have not been explored.

In summary, we found little evidence to support claims that 12-h shifts per se can solve or ameliorate staff shortages, with some evidence suggesting that turnover may be increased. The existing evidence on recruitment and retention seems to point to more complex mechanisms when it comes to shift length, with aspects such as flexibility, choice and preferences playing a crucial role.

## Discussion

In this paper, we have noted the many value propositions that have been made to support the introduction of 12-h shifts in nursing. We have explored the empirical evidence for these propositions and have found little, if any support. While direct measurement of efficiency is limited, the available evidence is consistent with 12-h shifts reducing efficiency. While there is an expressed preference for 12-h shifts in some quarters, it is unclear whether positive findings in relation to staff preference may result from the process of consultation in an attempt to meet staff needs. While there is some indication that the shift pattern may help to recruit staff under some circumstances, there is also evidence suggesting that retaining staff may become more problematic. Whether the turnover intention is increased because of the experience of working 12-h shifts or because staff who are attracted to this working pattern are less committed is unclear. It is possible that both mechanisms could operate simultaneously for different members of the workforce. Elements of preference and constraint cannot be ignored on the basis of evidence of negative effects alone, and the improvements that long shifts offer to work-life balance of many nurses cannot be lightly discarded. Forcing nurses to work shift patterns they do not want to or cannot work is likely to lead to dissatisfaction and high turnover.

### Limitations of current evidence and future research

We did not assess studies’ quality formally, but note that the majority of studies used to support the value propositions around 12-h shifts have small samples, and are either pilots, service evaluations or before-and-after studies where the level of staff involvement in the decision of introducing 12-h shifts is unclear. While the evidence that links harms to 12-h shifts relative to 8-h shifts is not without limitations, in that most studies rely on self-reported data with cross-sectional designs much of it has been conducted on a large scale with potentially generalisable samples comprising thousands of nursing staff. Nonetheless, the surveys often fail to include important shift work variables, including total number of hours worked per week and nurses’ preferences. For some outcomes, for example sickness absence, longitudinal designs and objective data mean that we can be more certain when drawing conclusions. Future studies should further explore the potential for improved shift patterns, considering nurses’ preferences and constraints and patient outcomes. Methods from the economics field, including discrete choice experiments are a promising way forward to inform change in nurses’ shift patterns. On the other hand, although giving staff choice and allowing mixed shift patterns within a given unit appears an attractive solution, the limited evidence does suggest that having a mixed shift pattern within one unit might be more resource intensive than deploying a single shift pattern within a unit.

### Limitations

Whilst this is a discussion paper and no formal review methods were applied, we have drawn on reviews undertaken with systematic methods to identify related evidence. While it is possible that other evidence exists, it seems unlikely that individual studies would contradict our fundamental conclusion: that the evidence for many of the value propositions for 12-h shifts is scant or absent.

## Conclusions

In conclusion, we found that the value propositions that have been advanced in favour of 12-h shifts in nursing are largely unsupported by evidence. While rarely conclusive, there is a considerable body of evidence pointing to direct risks to the quality of patient care and unintended consequences for staff wellbeing associated with longer shifts. Despite this, 12-h shifts persist.

While many of the direct economic arguments for implementing these long shifts based on efficiency and productivity claims should be abandoned, 12-h shifts cannot be simply discounted. Long shifts may indeed have a crucial role in delivering flexibility for both services and accommodating staff choice. If 12-h shifts are of value in supporting such flexibility the true costs of implementing them will have to be recognised and paid if adverse consequences for patients, staff and ultimately the health system itself, are to be avoided. Arriving at ‘ideal’ shift patterns may involve balancing competing risks for staff and patients and introducing appropriate mitigation to minimise adverse effect and maximise benefit. One such mitigation may require a recognition that increased use of 12-h shifts could require more, not less, staff.

## Supplementary Information

**Additional file 1: **Search strategy.

## Data Availability

Not applicable.
