# Supplementary material for: Because they’re worth it? A discussion paper on the value of 12-h shifts for hospital nursing
Source: Hum Resour Health. 2022 May 7;20:36. doi: 10.1186/s12960-022-00731-2 (PMC9077839; doi:10.1186/s12960-022-00731-2)
Supplement: Supplementary file 1 — Additional file 1: Search strategy. [file 12960_2022_731_MOESM1_ESM.docx]

| **Database** | **Search ID#** | **Search terms** | **Results** |
| --- | --- | --- | --- |
| EconLit | #1 | Labour supply | 41,082 |
|  | #2 | Job choice | 11,477 |
|  | #3 | Turnover | 10,495 |
|  | #4 | Recruitment | 1,914 |
|  | #5 | Retention | 2,585 |
|  | #6 | Productiv* | 158,082 |
|  | #7 | Efficien* | 131,438 |
|  | #8 | Sickness absence | 175 |
|  | #9 | Absenteeism | 651 |
|  | #10 | #1 OR #2 OR #3 OR #4 OR #5 OR #6 OR #7 OR #8 OR 9 | 310,366 |
|  | #11 | Nurs* | 2,224 |
|  | #12 | Shift work | 2,360 |
|  | #13 | Shift length | 193 |
|  | #14 | Long shift | 3,268 |
|  | #15 | Shift pattern | 2,155 |
|  | #16 | Schedul* | 8,609 |
|  | #17 | Roster* | 124 |
|  | #18 | #5 OR #6 OR #7 OR #8 OR #9 OR #10 | 15,729 |
|  | #19 | #6 AND #7 AND #14 | 50 |
|  | | | |
| CINAHL | #1 | Labour supply | 6,914 |
|  | #2 | Job choice | 1,677 |
|  | #3 | Turnover | 14,270 |
|  | #4 | Recruitment | 53,135 |
|  | #5 | Retention | 40,747 |
|  | #6 | Productiv* | 26,829 |
|  | #7 | Efficien* | 94,848 |
|  | #8 | Sickness absence | 1,947 |
|  | #9 | Absenteeism | 6,585 |
|  | #10 | #1 OR #2 OR #3 OR #4 OR #5 OR #6 OR #7 OR #8 OR 9 | 225,447 |
|  | #11 | (MM "Shiftwork") | 1,997 |
|  | #12 | Nurs* | 993,811 |
|  | #13 | Hospital | 544,194 |
|  | #14 | #10 AND #11 AND #12 AND #13 | 57 |
|  | | | |
| MEDLINE via OVID | #1 | labour supply.mp. | 84 |
|  | #2 | job choice.mp. | 49 |
|  | #3 | Personnel Turnover/ | 4572 |
|  | #4 | recruitment.mp. | 117,439 |
|  | #5 | productivity.mp. or exp Efficiency/ | 72,314 |
|  | #6 | exp Absenteeism/ | 5816 |
|  | #7 | #1 or #2 or #3 or #4 or #5 or #6 | 197,692 |
|  | #8 | Work Schedule / or Shift Work Schedule/ | 6521 |
|  | #9 | Nursing Staff, Hospital/ | 29,864 |
|  | #10 | #7 AND #8 AND #9 | 31 |
